# Coding, Recording and Incidence of Different Forms of Coronary Heart Disease in Primary Care

**DOI:** 10.1371/journal.pone.0029776

**Published:** 2012-01-19

**Authors:** Nawaraj Bhattarai, Judith Charlton, Caroline Rudisill, Martin C. Gulliford

**Affiliations:** 1 Department of Primary Care and Public Health Sciences, King's College London, London, United Kingdom; 2 Department of Social Policy and LSE Health, London School of Economics, London, United Kingdom; University of Modena and Reggio Emilia, Italy

## Abstract

**Objectives:**

To evaluate the coding, recording and incidence of coronary heart disease (CHD) in primary care electronic medical records.

**Methods:**

Data were drawn from the UK General Practice Research Database. Analyses evaluated the occurrence of 271 READ medical diagnostic codes, including categories for ‘Angina’, ‘Myocardial Infarction’, ‘Coronary Artery Bypass Grafting’ (CABG), ‘percutaneous transluminal coronary angioplasty’ (PCTA) and ‘Other Coronary Heart Disease’. Time-to-event analyses were implemented to evaluate occurrences of different groups of codes after the index date.

**Results:**

Among 300,020 participants aged greater than 30 years there were 75,197 unique occurrences of coronary heart disease codes in 24,244 participants, with 12,495 codes for incident events and 62,702 for prevalent events. Among incident event codes, 3,607 (28.87%) were for angina, 3,262 (26.11%) were for MI, 514 (4.11%) for PCTA, 161 (1.29%) for CABG and 4,951 (39.62%) were for ‘Other CHD’. Among prevalent codes, 20,254 (32.30%) were for angina, 3,644 (5.81%) for MI, 34,542 (55.09%) for ‘Other CHD’ and 4,262 (6.80%) for CABG or PCTA. Among 3,685 participants initially diagnosed exclusively with ‘Other CHD’ codes, 17.1% were recorded with angina within 5 years, 5.6% with myocardial infarction, 6.3% with CABG and 8.6% with PCTA. From 2000 to 2010, the overall incidence of CHD declined, as did the incidence of angina, but the incidence of MI did not change. The frequency of CABG declined, while PCTA increased.

**Conclusion:**

In primary care electronic records, a substantial proportion of coronary heart disease events are recorded with codes that do not distinguish between different clinical presentations of CHD. The results draw attention to the need to improve coding practice in primary care. The results also draw attention to the importance of code selection in research studies and the need for sensitivity analyses using different sets of codes.

## Introduction

Coronary Heart Disease (CHD) is a major cause of morbidity and mortality in the United Kingdom (UK); one in five men and one in seven women died from the disease in 2008 accounting for a total of 88,000 deaths [1]. CHD costs an estimated £9 billion a year to the UK economy, of which around 36% is due to direct health care costs, 43% is due to productivity losses and 21% is due to informal care for people with CHD [1]. In the past thirty years, CHD mortality rates have been falling [1]. Although CHD death rates have been falling at one of the fastest rates in Europe, mortality still remains relatively high in comparison to many Western European countries. In 2003, among the Western European countries, only Ireland and Germany had higher rates than UK [1]. The decline in mortality may be explained by a reduction in the major risk factors for CHD and a decrease in the occurrence of new major coronary events [2]. However, the decline in CHD mortality may be partly attributed to improvements in treatment and secondary prevention [3].

In Britain, patients with CHD are frequently diagnosed, given initial treatment and long-term preventive medical follow-up by general practitioners (GPs) in primary care [4], [5]. Patients may present in primary care with one of several clinical presentations of CHD including, principally, angina and myocardial infarction. If patients are admitted directly to specialist care as emergency cases then specific therapeutic interventions, including coronary artery bypass grafting and percutaneous transluminal coronary angioplasty, may sometimes be recorded in primary care as the first manifestation of CHD. Primary care practitioners are increasingly making use of clinical management recommendations that are intended to be applied to all of their patients with CHD [6], [7].

Primary care databases, including the General Practice Research Database (GPRD), provide anonymised data from large population-based samples of patients in primary care in England, Wales, Scotland and Northern Ireland [8]. Data from the GPRD have potential to provide important epidemiological data concerning the recent incidence and management of CHD in the UK population. Primary care data may also be used for aetiological epidemiological studies, health services and public health research, as well as for pharmaco-epidemiology and pharmacovigilance. The validity of diagnoses recorded in primary care databases has been well studied. A recent review suggested that diagnoses in GPRD are associated with very high predictive values [9]. Nevertheless, there may be difficulties in establishing case definitions in coded records from primary care. A recent study of stroke in GPRD found that there was substantial variation, between practices and over time, in the use of medical diagnostic codes for stroke. Investigators were sometimes uncertain of the significance of particular stroke codes, which could have distinct clinical and prognostic associations [10]. This study called for greater transparency in the selection of medical code sets that are used for case definitions in the analysis of electronic patient records, as well as for more open reporting and sharing of code sets.

The present study was implemented as part of a larger project to explore the incidence and mortality of cardiovascular diseases in GPRD. The objective of the present analysis was to explore the coding of coronary heart disease into primary care records, and to understand trends in the utilisation of CHD medical codes over time, in order to inform estimates of the incidence and prevalence of different manifestations of CHD.

## Methods

### General Practice Research Database

The General Practice Research Database (GPRD) is a large database of anonymised electronic medical records from primary care [11]. The GPRD has collected data since 1987. The GPRD currently includes data on 5 million active patients' use of primary care service from 625 primary care practices throughout the United Kingdom. The GPRD is representative of a demographic breakdown of the UK population [8]. Several studies, using a number of methods to assess the validity of medical diagnoses and information quality, have confirmed the validity of the GPRD estimates [9], [12].

### Medical code selection

Clinical events were initially recorded in GPRD using Oxford Medical Information Systems (OXMIS) codes but in more recent years READ terms have been used exclusively with OXMIS codes being mapped to their READ code equivalents [13]. We initially referred to the CHD diagnostic codes (324 codes) from *Key Health Statistics from General Practice* [13], we then compared these codes with ones generated from NHS Clinical Terminology browser, *Clinical Terms (the READ codes) version-3* [14]. This gave a total of 345 Read and OXMIS codes for CHD. After omitting OXMIS codes there were 271 READ codes. These were classified into five groups including 38 codes for ‘Angina’, 64 for ‘Myocardial Infarction’, 68 for ‘Coronary Artery Bypass Grafting’ (CABG) and 25 for ‘Percutaneous coronary transluminal angioplasty’ (PCTA). For the present analyses, codes for pre-infarction syndromes including unstable angina and acute coronary syndromes were grouped with ‘angina’. There were 76 codes for ‘Other Coronary Heart Disease’ including codes for non-specific terms including ‘Ischaemic heart disease’ and ‘coronary heart disease annual review’.

### Participants and data analysis

The study population consisted of a random sample of 300,020 participants, stratified by gender, who were aged >30 years of age and registered at a GPRD practice during the period 1st January 2004 to 30th June 2010. Participants had a minimum of 12 months of ‘up-to-standard’ follow-up calculated as the difference between the patient registration end date and registration start date. The last data collection date for the study participants was 25th October 2010.

We estimated incidence and prevalence of CHD by five categories for each year from 2000 to 2010. For these analyses, the start date was defined as the later of the patient's registration date or the practices ‘up to standard’ start date (the date on which the practices records were judged to be of a quality acceptable for research). The end date was the earliest of the death date, the end of registration date or the last data collection date. The index date was the first recorded occurrence of a CHD medical code. All medical codes recorded within 30 days of the index date were considered as incident codes, provided the index date was more than one year after the start date. Codes recorded more than 30 days after the index date, or within 12 months of the start date, were considered as prevalent codes. Rates for men and women were standardised to the European Standard Population. We implemented time-to-event analyses to explore the subsequent pattern of occurrence of codes in participants who were initially diagnosed with non-specific codes. The analysis started at the incidence date and ended at the occurrence of a specific code (failure) or end of record (censoring).

### Ethics

We utilised a fully anonymised data set from the General Practice Research Database. We did not obtain participant's consent because the participant data was taken from the fully anonymised data set and no participant's identity details were revealed. There was no need for participant consent. The research represents part of a study approved by the Independent Scientific Advisory Committee (ISAC) of the Medicines and Healthcare products Regulatory Agency (MHRA) (ISAC Protocol No. 09-085).

## Results

The sample included 300,020 participants who were registered with the GPRD between 2004 and 2010. Analysis of the participants' clinical and referral records identified 24,244 participants with 134,749 clinical or referral events associated with CHD codes. These events utilised 217 of the 271 codes that were included in the study. There were 54 Read codes for CHD that were not utilised in this sample, including 24 for CABG, 13 for MI, 11 for ‘Other CHD’, 3 for Angina and 3 for PCTA.

**Figure 1:**
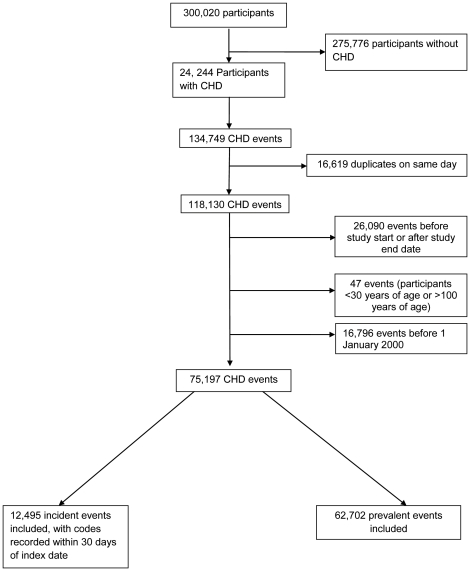
Flow chart showing the data extraction from the General Practice Research Database (GPRD).

Events were excluded if they were: before 1st January 2000 or the start date; after the end date; before 30 years; or after 100 years of age. Codes that were duplicated on the same date were also excluded (Figure 1). There were then 75,197 events for further analysis including 12,495 incident events and 62,702 prevalent events. The frequency of occurrence of individual codes by CHD category is shown in Table S1. Codes that were not utilised were omitted. Among incident codes, ‘Other CHD’ (39.62%) contributed the largest proportion, followed by angina (28.87%), MI (26.11%), PCTA (4.11%) and CABG (1.29%) (Table S1). Among the prevalent CHD codes, the order of frequency remained the same but ‘Other CHD’ contributed 55.09%, followed by angina (32.30%), MI (5.81%), PCTA (3.72%) and CABG (3.08%) in total (Table S1).

Table 1 shows the age standardised incidence rates for CHD by study year in men and women respectively with incidence rates grouped by CHD category. In men, there was an almost 30% decline in the incidence of all CHD in 2010 when compared to the incidence in the year 2000. Similarly, angina and other CHD incidence have fallen among men. The incidence of MI remained largely constant over the years. The PCTA/CABG showed largely constant incidence over the years in both genders. When compared with the males, lower incidences were observed in the females. The incidence of MI in females showed an uneven pattern. Overall, CHD incidence for women has fallen from 2000–2010 as for men, by about 30%.

**Table 1:** Incidence of new CHD diagnoses in adults aged 30 to 100 years in GPRD.

		Age-standardised incidence rate (per 1000 general population)				
Year	Number of participants	All CHD	Angina	CABG/PCTA	MI	Other CHD
**MEN**						
**2000**	432	5.39	2.29	0.20	1.42	1.47
**2001**	576	6.18	2.41	0.24	1.71	1.82
**2002**	619	5.80	2.27	0.15	1.65	1.73
**2003**	670	5.64	1.72	0.11	1.54	2.26
**2004**	672	5.21	1.36	0.20	1.59	2.05
**2005**	598	4.38	1.37	0.14	1.53	1.34
**2006**	609	4.28	1.14	0.26	1.46	1.42
**2007**	710	4.83	1.25	0.20	1.73	1.65
**2008**	644	4.28	1.00	0.23	1.64	1.41
**2009**	618	4.08	1.02	0.23	1.42	1.41
**2010**	422	3.86	0.79	0.25	1.53	1.30
**P value** a		<0.001	<0.001	0.223	0.750	0.108
**WOMEN**						
**2000**	284	3.00	1.66	0.03	0.53	0.77
**2001**	428	3.92	1.85	0.07	0.66	1.34
**2002**	483	3.91	1.82	0.06	0.57	1.46
**2003**	450	3.26	1.18	0.06	0.64	1.39
**2004**	455	3.02	0.98	0.10	0.54	1.40
**2005**	372	2.25	0.72	0.07	0.55	0.91
**2006**	398	2.33	0.71	0.06	0.72	0.83
**2007**	427	2.46	0.82	0.05	0.69	0.91
**2008**	349	1.86	0.53	0.12	0.49	0.73
**2009**	384	2.12	0.60	0.13	0.55	0.85
**2010**	234	1.85	0.55	0.09	0.61	0.61
**P value** a		0.001	<0.001	0.018	0.964	0.038

a test for linear trend.

Figures are age-standardised incidence rates per 1000 using the European Population as standard.

The overall rate of recording of codes for angina declined substantially in males from 17.79 per 1000 patient years in 2000 to 4.97 per 1000 patient years in 2010 and in females from 9.56 per 1000 patient years in 2000 to 2.55 per 1000 patient years in 2010 (Table 2). The rate of recording of codes for MI and CABG also follow a similar trend for both men and women. The rate of recording of codes for ‘Other CHD’ showed an increasing trend in men and women from year 2000 and peaked before beginning a declining trend in 2005 that continued to 2010. PCTA in males increased from 0.88 per 1000 patient years in 2000 to 2.04 per 1000 patient years in 2007 and again decreased 2008. Females showed an increase in PCTA from 0.31 per 1000 person years in 2000 to 0.52 per 1000 person years in 2010. The trend across both men and women is for CABG prevalence to be falling while PCTA prevalence has increased from 2000–2010.

**Table 2:** Rate of recording of different groups of CHD codes in adults aged 30 to 100 years in GPRD.

	Age-standardised recording rate (per 1000 general population)				
Year	Angina	MI	Other CHD	CABG	PCTA
**MEN**					
**2000**	17.79	4.06	14.42	1.76	0.88
**2001**	18.40	4.00	16.30	1.93	0.98
**2002**	17.99	4.27	17.15	1.77	1.29
**2003**	16.54	3.77	19.28	1.62	1.52
**2004**	11.42	3.59	21.96	1.78	1.62
**2005**	8.41	3.31	20.38	1.20	1.75
**2006**	7.79	2.97	19.28	0.81	1.79
**2007**	7.44	3.50	19.42	0.85	2.04
**2008**	6.14	3.38	17.41	0.78	1.68
**2009**	5.66	3.03	15.57	0.79	1.63
**2010**	4.97	2.80	15.04	0.69	1.48
**Pvalue**	<0.001	<0.001	0.958	<0.001	0.018
**WOMEN**					
**2000**	9.56	1.45	5.66	0.39	0.31
**2001**	10.50	1.39	7.71	0.56	0.22
**2002**	10.08	1.37	8.22	0.39	0.33
**2003**	8.18	1.62	8.89	0.36	0.44
**2004**	6.27	1.20	10.54	0.36	0.56
**2005**	5.01	1.04	9.25	0.12	0.61
**2006**	4.26	1.34	9.10	0.17	0.61
**2007**	3.74	1.29	8.68	0.18	0.50
**2008**	2.98	1.02	7.47	0.19	0.48
**2009**	2.90	1.11	7.03	0.14	0.45
**2010**	2.55	1.09	6.75	0.12	0.52
**Pvalue**	<0.001	0.014	0.929	<0.001	0.041

Figures are age-standardised recording rates per 1000 using the European Population as standard.

Table 3 shows the results of time to event analyses. Proportions were estimated from the failure function. Among 10,834 participants with incident CHD events, there were 7,093 whose incident code was not for angina. In these participants, the cumulative proportion with angina recorded over the subsequent five years was 19.4%. There were 7,635 participants not initially recorded with MI, of whom 6.2% were diagnosed with MI over the next five years. There were 7.4% coded with CABG and 11.7% with PCTA over a five year period. In 3,685 participants who were exclusively diagnosed with ‘Other CHD’ codes as incident events, the proportions who were subsequently recorded with specific categories of CHD were generally similar to those observed in the whole sample. The initial episode was defined using a 30 day time window, subsequent codes may include more specific designations of this initial episode as well as possible further clinical episodes of CHD in the same or different form.

**Table 3:** Occurrence of codes after the initial diagnosis of CHD.

		Angina	Myocardial Infarction	CABG	PCTA
All participants with incident CHD	10,834				
Number Included^a^		7,093	7,635	10,490	10,161
Cumulative proportion (%) diagnosed with specified form of CHD by year since diagnosis	1	11.1	3.9	5.2	9.4
	2	15.0	4.6	6.5	10.5
	3	16.9	5.1	7.0	11.0
	4	18.4	5.7	7.2	11.3
	5	19.4	6.2	7.4	11.7
Participants initially diagnosed with ‘Other CHD’ only	3,685				
Number included		3,685	3,685	3,685	3,685
Cumulative proportion (%) diagnosed with specified form of CHD by year since diagnosis	1	9.4	3.6	4.6	6.8
	2	13.1	4.3	5.5	7.5
	3	14.8	4.7	6.0	7.8
	4	16.3	5.3	6.1	8.2
	5	17.1	5.6	6.3	8.6

a participants initially diagnosed with the index condition were omitted.

## Discussion

### Main findings

This study analysed the medical codes used to record coronary heart disease in a large primary care database. The results show that a substantial proportion of CHD events, including consultations and referrals, are coded in primary care using terms that do not distinguish between angina and myocardial infarction. The frequency of recording of CHD codes has declined over time, consistent with a declining incidence of CHD. As this process has developed there has been a shift towards relatively greater use of non-specific terms to record CHD events. Among participants whose initial events are exclusively recorded using non-specific terms for ‘Other CHD’, only a minority have specific terms recorded over the subsequent five years of follow-up. Thus a substantial decline in the recording of angina may in part be artefactual as a result of an increase in the use of non-specific codes.

### Comparison with other studies

The present estimates for the incidence of angina in men (1.02 per 1000) and in women (0.60 per 1000) in the year 2009 are higher than the findings from another UK study [1] with angina incidence of 0.48 per 1000 in men and 0.28 per 1000 in women. However, unlike our study, this study included men and women of all ages. Age-specific rates or age-standardised rates for a specified age-range should be preferred for comparison. Consistent with other studies [2], [3], [15], our results show that CHD incidence and prevalence is declining in the United Kingdom for both men and women. There has also been a shift in treatment away from an invasive procedure (CABG) towards a higher usage rate of a less invasive procedure (PCTA). The overall reduction in CHD across the UK is largely due to reduced major risk factors and improvements in the widely used effective treatment [3]. The largest contributors to the decline of the risk factors came from the decline in cigarette smoking, non HDL cholesterol, HDL cholesterol, systolic blood pressure, and significant contribution from physical activity [3], [15]–[17]. Gender differences in CHD rates are consistent with data reported elsewhere [1].

### Strengths and Limitations

Participants with possible coronary heart disease were identified using diagnostic READ codes from the GPRD. GPRD practices all use VISION software, while other practice systems are also in use in the UK. It is likely that our findings are applicable to practices using other systems, but we note that some systems encourage greater reliance on free-text entries. We analysed data for a very large random sample from a database that covers approximately 6% of the UK population. All participants were registered at some time between 2004 and 2010, our estimates therefore did not include participants who presented with CHD and died before 2004. The CHD codes employed have been updated over time according to advancing understanding of angina and myocardial infarction as well as new recommendations for treatment. We acknowledge that the category of ‘Other CHD codes’ is not homogenous and might be divisible into other categories. For example, codes for several coronary artery operations that are not CABG are included in this category. These codes were used infrequently while the more frequently used codes in the ‘Other CHD’ category were clearly non-specific. We caution that our primary interest was in evaluating trends in the occurrence of different medical codes rather than the incidence of disease per se. Nevertheless, we believe our estimates are consistent with incidence results reported from other studies [2], [18].

### Conclusion

In primary care electronic records, a substantial proportion of coronary heart disease events are recorded with codes that do not distinguish between different clinical presentations of CHD. While CHD has declined in incidence over time, the use of less specific terms for diagnosis has shown a relative increase. These results draw attention to the need to improve coding practice primary care. In their day to day practice, general practitioners are able to draw on additional information including letters received from specialists, as well as free-text entries. Furthermore, many primary care interventions for CHD are relevant to all patients with the condition. For these reasons, making more precise code selections may have diminished relevance for primary care practitioners. Nevertheless, good recording is generally desirable in order to promote good clinical practice as well as to enhance the utility of coded records for researchers.

The present findings are of importance to researchers. Code sets for ‘angina’ or ‘myocardial infarction’ may have limited sensitivity for these conditions if substantial proportions are coded with ‘Other CHD’. More highly selected categories such as preinfarction syndromes may be associated with similar or greater difficulties. There is also a need to clarify whether certain groups of patients are more, or less, likely to be designated with certain codes, leading to enhanced potential for selection bias in constructing participant samples. These results therefore draw attention to the importance of code selection in research studies and the need for transparency in the reporting of case definitions, as well as the importance of sensitivity analyses using different sets of codes.

## Supporting Information

Table S1 **Shown here are the READ terms for CHD, READ Codes, Incident and Prevalent CHD codes frequency, and Disease type.**
(DOC)
