# Supplementary material for: Coding, Recording and Incidence of Different Forms of Coronary Heart Disease in Primary Care
Source: PLoS One. 2012 Jan 19;7(1):e29776. doi: 10.1371/journal.pone.0029776 (PMC3261876; doi:10.1371/journal.pone.0029776)
Supplement: Table S1 — Shown here are the READ terms for CHD, READ Codes, Incident and Prevalent CHD codes frequency, and Disease type. (DOC) [file pone.0029776.s001.doc]

**Table S1: READ terms for CHD, READ Codes, Incident and Prevalent CHD codes frequency, and Disease type**

| **READ Terms** | **READ Code** | **Incident CHD codes (12,495)** | | **Prevalent CHD codes (62,702)** | |  |
| --- | --- | --- | --- | --- | --- | --- |
|  |  | **Freq.** | **%** | **Freq.** | **%** |  |
|  |  |  |  |  |  |  |
| **Angina Codes** |  |  |  |  |  |  |
| Angina pectoris | G33..00 | 2658 | 20.85 | 11554 | 18.03 |  |
| Acute coronary syndrome | G311500 | 293 | 2.30 | 451 | 0.70 |  |
| Unstable angina | G311.13 | 75 | 0.60 | 494 | 0.79 |  |
| Unstable angina | G311100 | 134 | 1.07 | 679 | 1.08 |  |
| Angina control | 662K.00 | 83 | 0.65 | 2120 | 3.31 |  |
| H/O: angina pectoris | 14A5.00 | 79 | 0.62 | 564 | 0.88 |  |
| Angina control - good | 662K000 | 46 | 0.36 | 3067 | 4.79 |  |
| Stable angina | G33z700 | 44 | 0.35 | 164 | 0.26 |  |
| Angina on effort | G33z300 | 38 | 0.30 | 169 | 0.26 |  |
| Angina pectoris NOS | G33z.00 | 9 | 0.07 | 15 | 0.02 |  |
| Angina pectoris NOS | G33zz00 | 28 | 0.22 | 129 | 0.21 |  |
| Acute coronary insufficiency | G31y000 | 33 | 0.26 | 60 | 0.09 |  |
| Crescendo angina | G311.11 | 21 | 0.16 | 79 | 0.12 |  |
| Ischaemic chest pain | G33z400 | 15 | 0.12 | 17 | 0.03 |  |
| New onset angina | G33z600 | 11 | 0.09 | 1 | 0.00 |  |
| Worsening angina | G311400 | 10 | 0.08 | 138 | 0.22 |  |
| Angina at rest | G311.14 | 0 | 0.00 | 4 | 0.01 |  |
| Angina at rest | G311200 | 9 | 0.07 | 44 | 0.07 |  |
| Angina control - worsening | 662K300 | 5 | 0.04 | 168 | 0.26 |  |
| Angina control NOS | 662Kz00 | 4 | 0.03 | 91 | 0.14 |  |
| Angina control - poor | 662K100 | 3 | 0.02 | 107 | 0.17 |  |
| Angina control - improving | 662K200 | 2 | 0.02 | 89 | 0.14 |  |
| Post infarct angina | G33z500 | 2 | 0.02 | 9 | 0.01 |  |
| Prinzmetal's angina | G331.00 | 2 | 0.02 | 7 | 0.01 |  |
| Antianginal therapy | 8B27.00 | 1 | 0.01 | 5 | 0.01 |  |
| H/O: Angina in last year | 14AJ.00 | 1 | 0.01 | 3 | 0.00 |  |
| Myocardial infarction aborted | G311000 | 1 | 0.01 | 1 | 0.00 |  |
| Angina decubitus | G330.00 | 0 | 0.00 | 4 | 0.01 |  |
| Angina decubitus NOS | G330z00 | 0 | 0.00 | 1 | 0.00 |  |
| Nocturnal angina | G330000 | 0 | 0.00 | 13 | 0.02 |  |
| Refractory angina | G311300 | 0 | 0.00 | 5 | 0.01 |  |
| Syncope anginosa | G33z200 | 0 | 0.00 | 1 | 0.00 |  |
| Variant angina pectoris | G331.11 | 0 | 0.00 | 1 | 0.00 |  |
| *Angina Total* |  | *3607* | *28.87* | *20254* | *32.30* |  |
| **Myocardial Infarction Codes** |  |  |  |  |  |  |
| Acute myocardial infarction | G30..00 | 1209 | 9.48 | 1279 | 2.00 |  |
| MI - acute myocardial infarction | G30..15 | 765 | 6.00 | 839 | 1.31 |  |
| Acute non-ST segment elevation myocardial infarction | G307100 | 519 | 4.07 | 595 | 0.93 |  |
| Acute myocardial infarction NOS | G30z.00 | 210 | 1.65 | 270 | 0.42 |  |
| Acute ST segment elevation myocardial infarction | G30X000 | 199 | 1.56 | 110 | 0.17 |  |
| Inferior myocardial infarction NOS | G308.00 | 119 | 0.93 | 126 | 0.20 |  |
| Old myocardial infarction | G32..00 | 50 | 0.39 | 154 | 0.24 |  |
| Anterior myocardial infarction NOS | G301z00 | 41 | 0.32 | 21 | 0.03 |  |
| Acute anterolateral infarction | G300.00 | 17 | 0.13 | 17 | 0.03 |  |
| Other specified anterior myocardial infarction | G301.00 | 17 | 0.13 | 18 | 0.03 |  |
| Acute subendocardial infarction | G307.00 | 13 | 0.10 | 11 | 0.02 |  |
| Acute inferolateral infarction | G302.00 | 11 | 0.09 | 8 | 0.01 |  |
| ECG: myocardial infarction | 323..00 | 9 | 0.07 | 3 | 0.00 |  |
| Acute anteroseptal infarction | G301100 | 8 | 0.06 | 15 | 0.02 |  |
| Heart attack | G30..14 | 8 | 0.06 | 33 | 0.05 |  |
| Acute non-Q wave infarction | G307000 | 7 | 0.05 | 16 | 0.02 |  |
| Acute inferoposterior infarction | G303.00 | 6 | 0.05 | 2 | 0.00 |  |
| Posterior myocardial infarction NOS | G304.00 | 6 | 0.05 | 7 | 0.01 |  |
| Silent myocardial infarction | G30..17 | 6 | 0.05 | 0 | 0.00 |  |
| Coronary thrombosis | G30..12 | 5 | 0.04 | 7 | 0.01 |  |
| Personal history of myocardial infarction | G32..12 | 4 | 0.03 | 51 | 0.08 |  |
| Silent myocardial ischaemia | G344.00 | 4 | 0.03 | 0 | 0.00 |  |
| Acute septal infarction | G30y200 | 3 | 0.02 | 0 | 0.00 |  |
| Acute Q-wave infarct | G309.00 | 2 | 0.02 | 3 | 0.00 |  |
| Attack - heart | G30..11 | 2 | 0.02 | 1 | 0.00 |  |
| Cardiac rupture following myocardial infarction (MI) | G30..13 | 2 | 0.02 | 1 | 0.00 |  |
| Dressler's syndrome | G310.11 | 2 | 0.02 | 7 | 0.01 |  |
| Lateral myocardial infarction NOS | G305.00 | 2 | 0.02 | 6 | 0.01 |  |
| Other acute myocardial infarction | G30y.00 | 2 | 0.02 | 2 | 0.00 |  |
| Other acute myocardial infarction NOS | G30yz00 | 2 | 0.02 | 0 | 0.00 |  |
| Postoperative myocardial infarction | G38..00 | 2 | 0.02 | 4 | 0.01 |  |
| Subsequent myocardial infarction of inferior wall | G351.00 | 2 | 0.02 | 2 | 0.00 |  |
| Transient myocardial ischaemia | G31y300 | 2 | 0.02 | 6 | 0.01 |  |
| Acute anteroapical infarction | G301000 | 1 | 0.01 | 0 | 0.00 |  |
| Haemopericardium/current comp folow acut myocard infarct | G360.00 | 1 | 0.01 | 0 | 0.00 |  |
| Healed myocardial infarction | G32..11 | 1 | 0.01 | 0 | 0.00 |  |
| Mural thrombosis | G30A.00 | 1 | 0.01 | 11 | 0.02 |  |
| Thrombosis - coronary | G30..16 | 1 | 0.01 | 7 | 0.01 |  |
| True posterior myocardial infarction | G306.00 | 1 | 0.01 | 0 | 0.00 |  |
| Acute posterolateral myocardial infarction | G30B.00 | 0 | 0.00 | 1 | 0.00 |  |
| Acute transmural myocardial infarction of unspecif site | G30X.00 | 0 | 0.00 | 1 | 0.00 |  |
| Atrial septal defect/curr comp folow acut myocardal infarct | G361.00 | 0 | 0.00 | 1 | 0.00 |  |
| ECG: myocardial infarct NOS | 323Z.00 | 0 | 0.00 | 1 | 0.00 |  |
| Postmyocardial infarction syndrome | G310.00 | 0 | 0.00 | 1 | 0.00 |  |
| Postoperative transmural myocardial infarction inferior wall | G381.00 | 0 | 0.00 | 1 | 0.00 |  |
| Subsequent myocardial infarction | G35..00 | 0 | 0.00 | 4 | 0.01 |  |
| Subsequent myocardial infarction of anterior wall | G350.00 | 0 | 0.00 | 2 | 0.00 |  |
| *MI Total* | *Total* | ***3262*** | *26.11* | ***3644*** | *5.81* |  |
| **Other CHD Codes** |  |  |  |  |  |  |
| Ischaemic heart disease | G3...00 | 1973 | 15.47 | 6426 | 10.03 |  |
| IHD - Ischaemic heart disease | G3...13 | 706 | 5.54 | 3260 | 5.09 |  |
| Coronary heart disease annual review | 6A2..00 | 694 | 5.44 | 16744 | 26.13 |  |
| Aspirin prophylaxis - IHD | 8B63.11 | 512 | 4.02 | 1978 | 3.09 |  |
| Coronary artery disease | G340.12 | 253 | 1.98 | 588 | 0.92 |  |
| Coronary heart disease review | 6A4..00 | 188 | 1.47 | 2554 | 3.99 |  |
| Ischaemic heart disease NOS | G3z..00 | 171 | 1.34 | 316 | 0.49 |  |
| Coronary artery operations | 792..00 | 46 | 0.36 | 348 | 0.54 |  |
| Coronary atherosclerosis | G340.00 | 40 | 0.31 | 46 | 0.07 |  |
| Single coronary vessel disease | G340000 | 39 | 0.31 | 122 | 0.19 |  |
| H/O: cardiovascular disease | 14A..00 | 38 | 0.30 | 114 | 0.18 |  |
| Triple vessel disease of the heart | G340.11 | 34 | 0.27 | 233 | 0.36 |  |
| Coronary heart disease medication review | 8B3k.00 | 31 | 0.24 | 435 | 0.68 |  |
| Double coronary vessel disease | G340100 | 31 | 0.24 | 96 | 0.15 |  |
| Coronary arteriograph.abnormal | 5543 | 30 | 0.24 | 120 | 0.19 |  |
| Open angioplasty of coronary artery | 7927500 | 24 | 0.19 | 122 | 0.19 |  |
| Coronary artery spasm | G332.00 | 16 | 0.13 | 22 | 0.03 |  |
| Coronary artery bypass graft occlusion | SP07600 | 15 | 0.12 | 118 | 0.18 |  |
| Endarterectomy of coronary artery NEC | 792B000 | 11 | 0.09 | 11 | 0.02 |  |
| [X]Ischaemic heart diseases | Gyu3.00 | 10 | 0.08 | 22 | 0.03 |  |
| Cardiac syndrome X | G37..00 | 10 | 0.08 | 46 | 0.07 |  |
| [V]Presence of coronary artery bypass graft - CABG | ZV45K11 | 9 | 0.07 | 103 | 0.16 |  |
| ECG: myocardial ischaemia | 322..00 | 8 | 0.06 | 9 | 0.01 |  |
| Atherosclerotic heart disease | G3...12 | 7 | 0.05 | 103 | 0.16 |  |
| Other chronic ischaemic heart disease | G34..00 | 7 | 0.05 | 10 | 0.02 |  |
| Asymptomatic coronary heart disease | G34z000 | 6 | 0.05 | 60 | 0.09 |  |
| Ischaemic cardiomyopathy | G343.00 | 6 | 0.05 | 24 | 0.04 |  |
| [V]Presence of coronary artery bypass graft | ZV45K00 | 5 | 0.04 | 59 | 0.09 |  |
| Preinfarction syndrome | G311.00 | 5 | 0.04 | 23 | 0.04 |  |
| Arteriosclerotic heart disease | G3...11 | 3 | 0.02 | 7 | 0.01 |  |
| Atherosclerotic cardiovascular disease | G342.00 | 3 | 0.02 | 2 | 0.00 |  |
| Attends coronary heart disease monitoring | 9Ob0.00 | 2 | 0.02 | 186 | 0.29 |  |
| Chronic myocardial ischaemia | G34y100 | 2 | 0.02 | 7 | 0.01 |  |
| H/O: myocardial infarct <60 | 14A3.00 | 2 | 0.02 | 84 | 0.13 |  |
| Other acute and subacute ischaemic heart disease NOS | G31yz00 | 2 | 0.02 | 1 | 0.00 |  |
| Ventricular cardiac aneurysm | G341000 | 2 | 0.02 | 11 | 0.02 |  |
| [V]Presence of aortocoronary bypass graft | ZV45700 | 1 | 0.01 | 1 | 0.00 |  |
| [V]Presence of coronary angioplasty implant and graft | ZV45800 | 1 | 0.01 | 20 | 0.03 |  |
| Coronary artery operations NOS | 792z.00 | 1 | 0.01 | 26 | 0.04 |  |
| Diagnostic transluminal operations on coronary artery | 792A.00 | 1 | 0.01 | 4 | 0.01 |  |
| H/O: myocardial infarct >60 | 14A4.00 | 1 | 0.01 | 48 | 0.07 |  |
| Other cardiac wall aneurysm | G341100 | 1 | 0.01 | 0 | 0.00 |  |
| Other chronic ischaemic heart disease NOS | G34z.00 | 1 | 0.01 | 1 | 0.00 |  |
| Other specified ischaemic heart disease | G3y..00 | 1 | 0.01 | 9 | 0.01 |  |
| Repair of aneurysm of coronary artery | 7927100 | 1 | 0.01 | 3 | 0.00 |  |
| Repair of coronary artery NEC | 792B.00 | 1 | 0.01 | 0 | 0.00 |  |
| Aneurysm of coronary vessels | G341200 | 0 | 0.00 | 1 | 0.00 |  |
| Aneurysm of heart | G341.00 | 0 | 0.00 | 3 | 0.00 |  |
| Aneurysm of heart NOS | G341z00 | 0 | 0.00 | 2 | 0.00 |  |
| Cardiac aneurysm | G341.11 | 0 | 0.00 | 1 | 0.00 |  |
| Chronic coronary insufficiency | G34y000 | 0 | 0.00 | 1 | 0.00 |  |
| Diagnostic transluminal operation on coronary artery NOS | 792Az00 | 0 | 0.00 | 1 | 0.00 |  |
| Diagnostic transluminal operation on coronary artery OS | 792Ay00 | 0 | 0.00 | 1 | 0.00 |  |
| Exploration of coronary artery | 7927400 | 0 | 0.00 | 2 | 0.00 |  |
| H/O: Myocardial infarction in last year | 14AH.00 | 0 | 0.00 | 1 | 0.00 |  |
| Intravascular ultrasound of coronary artery | 792A100 | 0 | 0.00 | 1 | 0.00 |  |
| Other acute and subacute ischaemic heart disease | G31..00 | 0 | 0.00 | 3 | 0.00 |  |
| Other acute and subacute ischaemic heart disease | G31y.00 | 0 | 0.00 | 1 | 0.00 |  |
| Other open operation on coronary artery NOS | 7927z00 | 0 | 0.00 | 2 | 0.00 |  |
| *Other CHD Total* | *Total* | ***4951*** | *39.62* | ***34542*** | *55.09* |  |
| **CABG Codes** |  |  |  |  |  |  |
| Coronary artery bypass graft operations | 792..11 | 86 | 0.67 | 1098 | 1.71 |  |
| Saphenous vein graft replacement of coronary artery OS | 7920y00 | 34 | 0.27 | 383 | 0.60 |  |
| Saphenous vein graft replacement of three coronary arteries | 7920200 | 8 | 0.06 | 94 | 0.15 |  |
| Saphenous vein graft replacement of four+ coronary arteries | 7920300 | 6 | 0.05 | 38 | 0.06 |  |
| Saphenous vein graft replacement of two coronary arteries | 7920100 | 6 | 0.05 | 48 | 0.07 |  |
| Saphenous vein graft replacement of coronary artery | 7920 | 5 | 0.04 | 53 | 0.08 |  |
| [V]Status following coronary angioplasty NOS | ZV45L00 | 4 | 0.03 | 23 | 0.04 |  |
| Saphenous vein graft replacement of one coronary artery | 7920000 | 3 | 0.02 | 17 | 0.03 |  |
| LIMA single anastomosis | 7925311 | 2 | 0.02 | 1 | 0.00 |  |
| Saphenous vein graft bypass of coronary artery | 7920.11 | 2 | 0.02 | 45 | 0.07 |  |
| Connection of other thoracic artery to coronary artery | 7926 | 1 | 0.01 | 0 | 0.00 |  |
| Other autograft bypass of coronary artery | 7921.11 | 1 | 0.01 | 9 | 0.01 |  |
| Other bypass of coronary artery NOS | 792Dz00 | 1 | 0.01 | 10 | 0.02 |  |
| Other specified other bypass of coronary artery | 792Dy00 | 1 | 0.01 | 5 | 0.01 |  |
| Other therapeutic transluminal operations on coronary artery | 7929 | 1 | 0.01 | 9 | 0.01 |  |
| Allograft bypass of coronary artery | 7922.11 | 0 | 0.00 | 7 | 0.01 |  |
| Allograft replacement of two coronary arteries | 7922100 | 0 | 0.00 | 1 | 0.00 |  |
| Autograft replacement of four of more coronary arteries NEC | 7921300 | 0 | 0.00 | 3 | 0.00 |  |
| Autograft replacement of one coronary artery NEC | 7921000 | 0 | 0.00 | 1 | 0.00 |  |
| Autograft replacement of three coronary arteries NEC | 7921200 | 0 | 0.00 | 8 | 0.01 |  |
| Connection of mammary artery to coronary artery | 7925 | 0 | 0.00 | 2 | 0.00 |  |
| Connection of mammary artery to coronary artery NOS | 7925z00 | 0 | 0.00 | 2 | 0.00 |  |
| LIMA sequential anastomosis | 7925011 | 0 | 0.00 | 1 | 0.00 |  |
| Other bypass of coronary artery | 792D.00 | 0 | 0.00 | 13 | 0.02 |  |
| Creation of bypass from mammary artery to coronary artery | 7925.11 | 0 | 0.00 | 1 | 0.00 |  |
| Double anastomosis of mammary arteries to coronary arteries | 7925000 | 0 | 0.00 | 1 | 0.00 |  |
| Other autograft replacement of coronary artery | 7921 | 0 | 0.00 | 18 | 0.03 |  |
| Other autograft replacement of coronary artery NOS | 7921z00 | 0 | 0.00 | 1 | 0.00 |  |
| Other replacement of coronary artery | 792C.00 | 0 | 0.00 | 1 | 0.00 |  |
| Other specified operations on coronary artery | 792y.00 | 0 | 0.00 | 7 | 0.01 |  |
| Prosthetic bypass of coronary artery | 7923.11 | 0 | 0.00 | 2 | 0.00 |  |
| Prosthetic replacement of coronary artery | 7923 | 0 | 0.00 | 1 | 0.00 |  |
| Revision of bypass for coronary artery | 7924 | 0 | 0.00 | 7 | 0.01 |  |
| Revision of bypass for two coronary arteries | 7924100 | 0 | 0.00 | 1 | 0.00 |  |
| Saphenous vein graft replacement coronary artery NOS | 7920z00 | 0 | 0.00 | 16 | 0.02 |  |
| Single anast mammary art to left ant descend coronary art | 7925200 | 0 | 0.00 | 3 | 0.00 |  |
| *CABG Total* | *Total* | ***161*** | *1.29* | ***1930*** | *3.08* |  |
| **PCTA Codes** |  |  |  |  |  |  |
| Transluminal balloon angioplasty of coronary artery | 7928 | 193 | 1.51 | 923 | 1.44 |  |
| Insertion of coronary artery stent | 7929400 | 176 | 1.38 | 782 | 1.22 |  |
| Perc translumin balloon angioplasty stenting coronary artery | 793G.00 | 73 | 0.57 | 269 | 0.42 |  |
| Percutaneous balloon coronary angioplasty | 7928.11 | 16 | 0.13 | 97 | 0.15 |  |
| Transluminal balloon angioplasty of coronary artery NOS | 7928z00 | 11 | 0.09 | 63 | 0.10 |  |
| Percut transluminal balloon angioplasty one coronary artery | 7928000 | 10 | 0.08 | 42 | 0.07 |  |
| Percutaneous transluminal laser coronary angioplasty | 7929000 | 8 | 0.06 | 34 | 0.05 |  |
| Insertion of drug-eluting coronary artery stent | 7929500 | 6 | 0.05 | 33 | 0.05 |  |
| Percut transluminal coronary thrombolysis with streptokinase | 7929100 | 6 | 0.05 | 1 | 0.00 |  |
| Rotary blade coronary angioplasty | 7929300 | 5 | 0.04 | 38 | 0.06 |  |
| Perc translum ball angio insert 1-2 drug elut stents cor art | 793G000 | 3 | 0.02 | 13 | 0.02 |  |
| Percut translum balloon angioplasty mult coronary arteries | 7928100 | 3 | 0.02 | 15 | 0.02 |  |
| Other therapeutic transluminal op on coronary artery NOS | 7929z00 | 1 | 0.01 | 1 | 0.00 |  |
| Perc translum balloon angioplasty stenting coronary art NOS | 793Gz00 | 1 | 0.01 | 6 | 0.01 |  |
| Percutaneous cor balloon angiop 3 more stents cor art NEC | 793G300 | 1 | 0.01 | 1 | 0.00 |  |
| Transluminal balloon angioplasty of coronary artery OS | 7928y00 | 1 | 0.01 | 6 | 0.01 |  |
| OS perc translumina balloon angioplast stenting coronary art | 793Gy00 | 0 | 0.00 | 1 | 0.00 |  |
| Other therapeutic transluminal op on coronary artery OS | 7929y00 | 0 | 0.00 | 1 | 0.00 |  |
| Perc tran ball angio ins 3 or more drug elut stents cor art | 793G100 | 0 | 0.00 | 1 | 0.00 |  |
| Perc translum balloon angioplasty insert 1-2 stents cor art | 793G200 | 0 | 0.00 | 4 | 0.01 |  |
| Percut translum balloon angioplasty bypass graft coronary art | 7928200 | 0 | 0.00 | 1 | 0.00 |  |
| *PCTA Total* |  | *514* | *4.11* | *2332* | *3.72* |  |
|  | | | | | | |
